# Vitamin E Mitigates Polystyrene-Nanoplastic-Induced Visual Dysfunction in Zebrafish Larvae

**DOI:** 10.3390/ijms26031216

**Published:** 2025-01-30

**Authors:** Febriyansyah Saputra, Azzah Dyah Pramata, Agoes Soegianto, Shao-Yang Hu

**Affiliations:** 1 Department of Biology, Faculty Sciences and Technology, Universitas Airlangga, Campus C, Surabaya 60115, Indonesia; ryansaputra252@gmail.com; 2 Department of Materials and Metallurgical Engineering, Faculty of Industrial Technology and Systems Engineering, Institut Teknologi Sepuluh Nopember, Surabaya 60116, Indonesia; azzah@its.ac.id; 3 Department of Biological Science and Technology, National Pingtung University of Science and Technology, Pingtung 912301, Taiwan

**Keywords:** polystyrene nanoplastics, vitamin E, visual dysfunction, oxidative stress, zebrafish

## Abstract

Vitamin E (VitE), a potent antioxidant, has demonstrated significant potential in mitigating oxidative stress and cellular damage, making it a valuable agent for countering environmental toxicities, including those caused by polystyrene nanoplastics (PSNPs). This study examined the effects of PSNPs on the zebrafish visual system and evaluated the protective role of VitE. Zebrafish embryos were exposed to PSNPs (0.01, 0.1, 1, and 10 μg/mL) with or without 20 μM VitE co-treatment from fertilization to 6 days post-fertilization (dpf). Visual function, morphology, and molecular responses were assessed at 4 or 6 dpf. Exposure to PSNPs at concentrations of 0.1 to 10 μg/mL significantly increased bioaccumulation in the zebrafish eye in a concentration-dependent manner and disrupted the visual system. These disruptions caused a reduction in the eye-to-body length ratio and decreased optomotor response positivity and swimming distance, indicating impaired visual function and behavior. Furthermore, PSNPs elevated reactive oxygen species (ROS) levels, induced retinal apoptosis, and disrupted gene expression related to visual development (*six6*, *pax2*, *pax6a*, and *pax6b*), apoptosis (*tp53*, *casp3*, *bax*, and *bcl2a*), and antioxidant defense (*sod1*, *cat*, and *gpx1a*). VitE co-treatment significantly mitigated these adverse effects, reducing oxidative damage, restoring antioxidant defenses, and preserving retinal function. This study highlights the potential of VitE as a protective agent against PSNP-induced visual dysfunction and underlines the urgent need to address nanoplastic pollution to protect aquatic ecosystems.

## 1. Introduction

VitE, a potent lipid-soluble antioxidant, has been extensively studied for its ability to counteract oxidative stress and protect against cellular damage [1]. It exists in two major forms: tocopherols, which are well studied and more abundant, and tocotrienols, a less understood and less widespread form of VitE that has also shown potential in modulating oxidative stress and inflammation [2]. Both forms have distinct roles in maintaining cellular homeostasis, particularly in tissues with high oxidative metabolism, such as the retina [3]. In ocular health, VitE plays a critical role in reducing ROS levels, restoring antioxidant defense mechanisms, and preventing apoptosis [4]. It has been shown to reduce radiation-induced cataract formation in rats, protect against retinal ischemia–reperfusion injury, and mitigate retinal damage caused by environmental toxins [2,3,5]. The natural abundance of VitE in ocular tissues highlights its essential role in preserving the integrity of the visual system [3]. Despite these established benefits, its potential to mitigate nanoplastic-induced visual dysfunction remains largely unexplored.

Nanoplastics (NPs) have emerged as a major environmental concern due to their pervasive distribution and significant toxicological effects, especially in aquatic ecosystems [6,7]. Among the most widely used plastics, polystyrene (PS) is a major contributor to the accumulation of micro- and nanoparticles in the environment [8,9]. These particles are known to bioaccumulate in aquatic organisms, disrupt cellular processes, and induce oxidative stress, posing a substantial threat to environmental health and ecosystem stability [6,10,11]. PSNPs readily infiltrate aquatic ecosystems, triggering oxidative stress, amplifying existing oxidative reactions, and disrupting cellular homeostasis [12]. Upon entering the bloodstream, PSNPs quickly cross critical barriers like the blood–brain barrier [13], resulting in DNA damage, mutagenic alterations, and cytotoxic effects [14,15]. In zebrafish, PSNP exposure has been associated with neurotoxicity and oxidative stress [16], decreased heart rate and locomotory inhibition [17], developmental abnormalities [13], disrupted glucose metabolism, altered cortisol levels, and potential behavioral changes [18]. However, while their systemic effects are increasingly understood, the specific impacts of PSNPs on the visual system remain poorly investigated, presenting a critical gap in PSNP toxicology research.

The eye is particularly vulnerable to oxidative stress [19], where an imbalance between oxygen-derived free radicals and antioxidants can lead to various degenerative diseases [20]. This vulnerability, combined with the retina’s high metabolic activity, makes the visual system a prime target for toxic insults [21]. Zebrafish (*Danio rerio*) has become a pivotal model in ocular toxicology due to its anatomical and functional similarities to the human eye, rapid development, and genetic tractability [22,23]. Outside of chemical toxicology, zebrafish studies focused on eyes are also adding to our understanding of radiation toxicity [24,25], the pathology of ocular diseases [26,27,28,29,30], and retinal genetics and regeneration [31,32]. Additionally, zebrafish are widely used in research aimed at understanding toxicity mechanisms, disease progression, and evaluating the effectiveness of various interventions [33,34,35]. Their capacity to mimic complex human eye conditions, coupled with their rapid lifecycle [21], makes zebrafish an invaluable model for studying visual system responses to toxicants and other environmental challenges.

This study investigates the toxic effects of PSNPs on the zebrafish visual system, focusing on oxidative stress, retinal damage, and gene expression changes. Furthermore, we examined the role of VitE in mitigating these effects. By demonstrating the protective role of VitE, this research provides foundational insights into potential strategies for managing the harmful impacts of NPs in controlled aquatic environments, such as aquaculture systems. While direct application in natural ecosystems may face challenges, these findings deepen our understanding of NP toxicity and highlight a promising approach to addressing the environmental and biological challenges posed by NP pollution.

## 2. Results

### 2.1. Bioaccumulation of PSNPs in Zebrafish Eye

**Figure 1:**
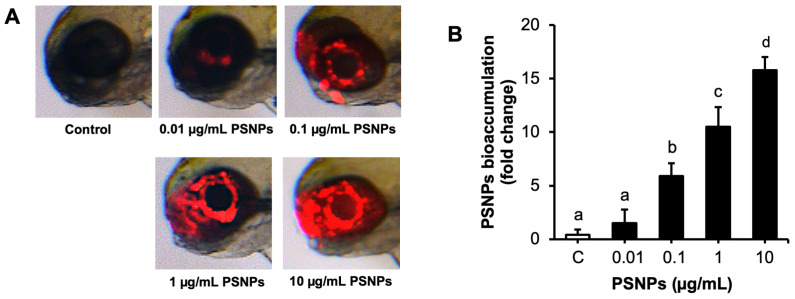
Bioaccumulation of fluorescence PSNPs on zebrafish eyes. (**A**) Representative images showing the bioaccumulation of PSNPs in zebrafish eyes at 96 hpf, visualized through red fluorescence, following exposure to control, 0.01, 0.1, 1, and 10 µg/mL PSNPs. (**B**) Quantification of PSNP bioaccumulation in zebrafish eyes exposed to control, 0.01, 0.1, 1, and 10 µg/mL PSNPs at 96 hpf. Data are presented as mean ± SD (*n* = 10 per group). Significant differences are denoted by different letters (*p* < 0.05). Each experiment was independently replicated three times.

The bioaccumulation of PSNPs was evaluated in zebrafish eyes after 96 hpf exposure to red fluorescently labeled PSNPs, with a focus on the eye region (Figure 1A). Fluorescence imaging demonstrated a clear bioaccumulation of PSNPs in the eye, with fluorescence intensity increasing with PSNP concentrations. However, the trend plateaued at higher concentrations, suggesting a potential saturation effect. At concentrations ranging from 0.1 to 10 µg/mL, fluorescence intensity was higher compared to both the control group and lower concentrations, indicating substantial PSNP bioaccumulation in the eye region. The quantitative analysis of PSNP bioaccumulation intensity further confirmed these findings, demonstrating a concentration-dependent pattern in PSNP bioaccumulation within the zebrafish eye. Statistical analysis revealed significant differences in PSNP bioaccumulation intensity among the exposure groups, with the highest concentration (10 µg/mL) showing the most pronounced bioaccumulation (Figure 1B). Additionally, the addition of VitE did not affect the bioaccumulation of PSNPs in the eye.

### 2.2. Effects of PSNP and VitE Addition on the Visual System

**Figure 2:**
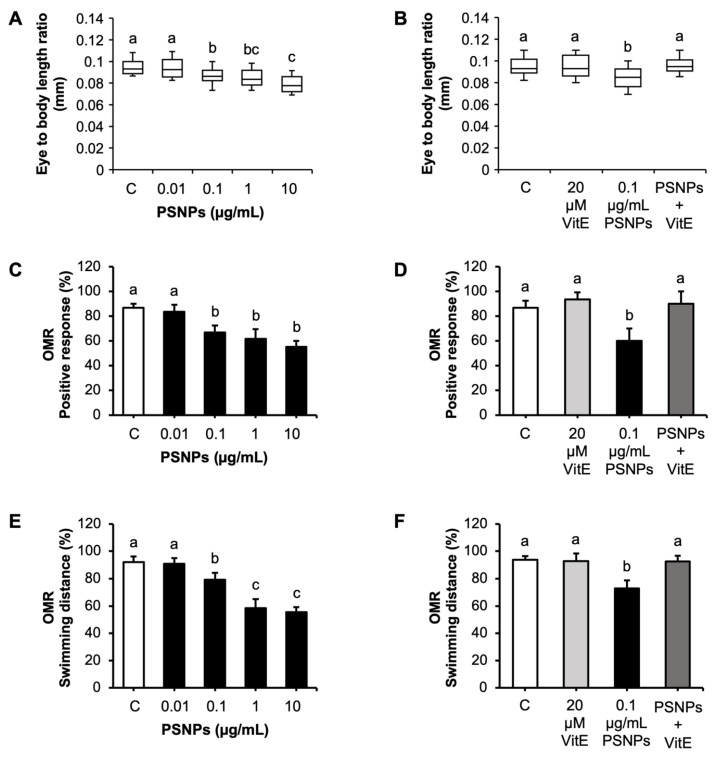
Effects of PSNP and VitE addition on visual system. (**A**) Eye-to-body length ratio in zebrafish at 96 hpf following exposure to control, 0.01, 0.1, 1, and 10 µg/mL. (**B**) Eye-to-body length ratio in zebrafish exposed to 0.1 µg/mL PSNPs with or without 20 µM VitE. (**C**) OMR positive response at 6 dpf in zebrafish exposed to control, 0.01, 0.1, 1, and 10 µg/mL PSNPs. (**D**) OMR positive response in zebrafish exposed to 0.1 µg/mL PSNPs with or without 20 µM VitE. (**E**) Swimming distance during OMR tests in zebrafish exposed to control, 0.01, 0.1, 1, and 10 µg/mL PSNPs. (**F**) Swimming distance during OMR tests in zebrafish exposed to 0.1 µg/mL PSNPs with or without 20 µM VitE. Data are presented as mean ± SD (*n* = 10 per group). Significant differences are denoted by different letters (*p* < 0.05). Each experiment was independently replicated three times.

To investigate the adverse effects of PSNPs on the visual system, zebrafish embryos were exposed to increasing concentrations of PSNPs (0.01, 0.1, 1, and 10 µg/mL) with or without 20 µM VitE. PSNP exposure at concentrations of 0.1 to 10 µg/mL significantly reduced the eye-to-body length ratio in zebrafish larvae (Figure 2A). However, the addition of VitE fully reversed this effect, restoring the eye-to-body length ratio to control levels (Figure 2B). Similarly, PSNP exposure at the same concentrations significantly decreased the percentage of larvae demonstrating a positive optomotor response (OMR) at 6 dpf (Figure 2C), and the addition of VitE fully reversed this impairment, restoring the OMR positive response to control levels (Figure 2D). Further analysis revealed that PSNP exposure significantly reduced swimming distance during OMR tests at 6 dpf, particularly at concentrations of 0.1 to 10 µg/mL (Figure 2E). The addition of VitE effectively mitigated this effect, restoring swimming distance to levels comparable to that of the control group (Figure 2F).

### 2.3. Effects of PSNP and VitE Addition on Retinal Apoptosis

**Figure 3:**
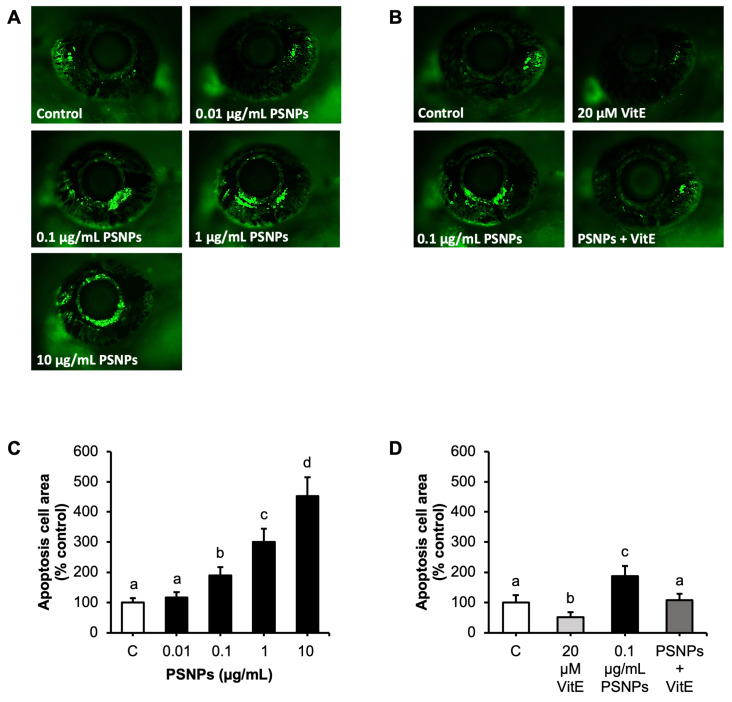
Effects of PSNP and VitE addition on apoptosis in the zebrafish retina at 96 hpf. (**A**) Representative images of apoptotic cells in the retina using AO staining, with apoptotic cells indicated by green fluorescence. Zebrafish were exposed to control, 0.01, 0.1, 1, and 10 µg/mL PSNPs. (**B**) Representative images of apoptotic cells in the retina of zebrafish exposed to 0.1 µg/mL PSNPs with or without 20 µM VitE. (**C**) Quantification of apoptotic cell area in the retina of zebrafish exposed to control, 0.01, 0.1, 1, and 10 µg/mL PSNPs. (**D**) Quantification of apoptotic cell area in zebrafish retina exposed to 0.1 µg/mL PSNPs with or without 20 µM VitE. Data are presented as mean ± SD (*n* = 10 per group). Significant differences are denoted by different letters (*p* < 0.05). Each experiment was independently replicated three times.

Retinal apoptosis was evaluated using acridine orange (AO) staining, where increased fluorescence in the GFP channel indicated elevated levels of apoptotic cell death. Zebrafish exposed to PSNPs at concentrations ranging from 0.1 to 10 µg/mL showed a concentration-dependent increase in the number of AO-positive cells (Figure 3A) and the area of apoptotic cells in the retina at 96 hpf (Figure 3C). Higher PSNP concentrations (1 to 10 µg/mL) resulted in significantly elevated apoptosis levels compared to controls. Notably, the addition of 20 µM VitE effectively reversed the apoptosis induced by 0.1 µg/mL PSNP exposure, as demonstrated in Figure 3B and 3D.

### 2.4. Effects of PNSP and VitE Addition on ROS Production in Retina

**Figure 4:**
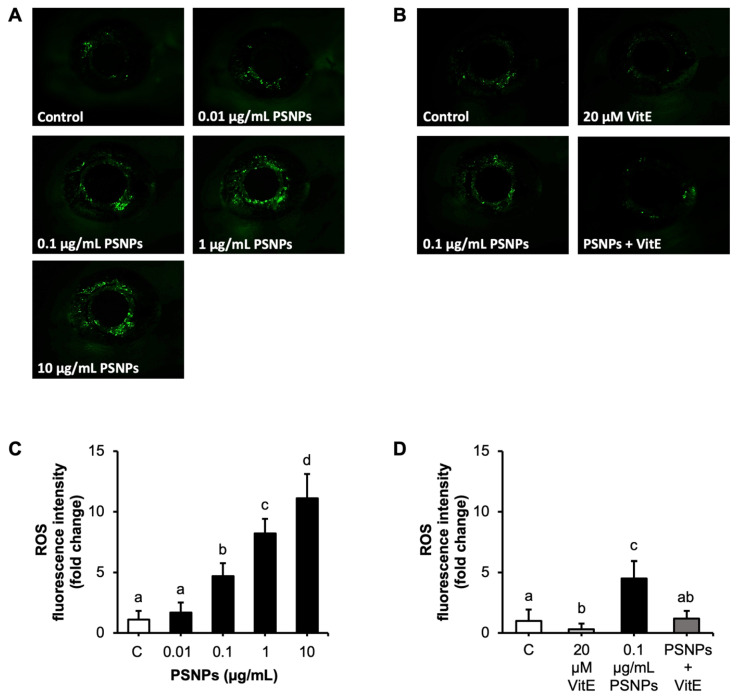
Effects of PSNP and VitE addition on ROS production in the zebrafish retina at 96 hpf. (**A**) Representative images of ROS production in the retina visualized using CM-H2DCFDA staining, with ROS production highlighted by green fluorescence. Zebrafish were exposed to control, 0.01, 0.1, 1, and 10 µg/mL PSNPs. (**B**) Representative images of ROS production in zebrafish retina exposed to 0.1 µg/mL PSNPs with or without 20 µM VitE. (**C**) Quantification of ROS production in zebrafish retina exposed to control, 0.01, 0.1, 1, and 10 µg/mL PSNPs. (**D**) Quantification of ROS production in zebrafish retina exposed to 0.1 µg/mL PSNPs with or without 20 µM VitE. Data are presented as mean ± SD (*n* = 10 per group). Significant differences are denoted by different letters (*p* < 0.05). Each experiment was independently replicated three times.

ROS production in the retina was assessed using CM-H2DCFDA staining, where green fluorescence indicates ROS accumulation. In the control group, minimal fluorescence was detected, reflecting low ROS levels. In contrast, zebrafish larvae exposed to PSNPs showed a significant, concentration-dependent increase in retinal ROS fluorescence intensity (Figure 4A). Quantitative analysis showed that exposure to PSNPs at concentrations of 0.1, 1, and 10 µg/mL significantly elevated ROS levels at 96 hpf, with the highest intensity observed at 10 µg/mL (Figure 4C) demonstrating that PSNPs induced oxidative stress in a concentration-dependent manner. Notably, the addition of VitE completely mitigated this increase in ROS production, as shown in Figure 4B,D.

### 2.5. Effects of PSNP and VitE Addition on Gene Expression

**Figure 5:**
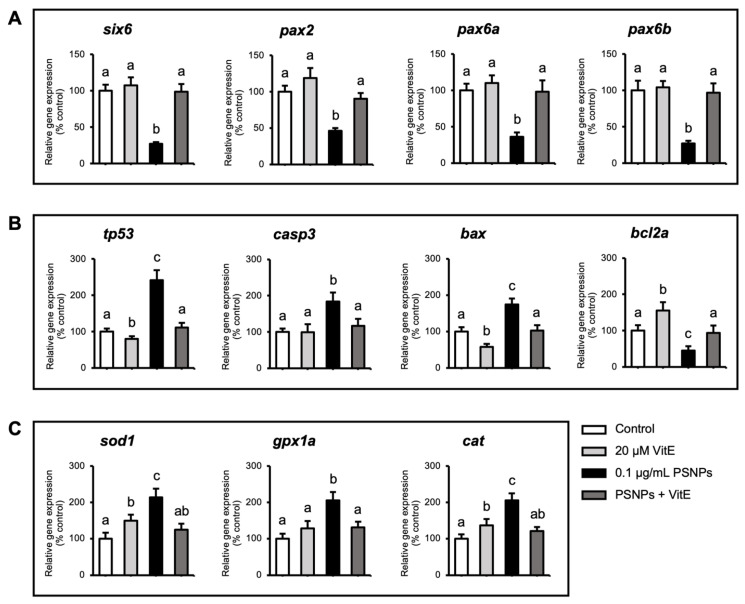
Effects of PSNP and VitE addition on gene expression in zebrafish at 96 hpf. Quantitative PCR analysis of gene expression was conducted on zebrafish exposed to 0.1 µg/mL PSNPs with or without 20 µM VitE. (**A**) Expression levels of genes related to visual development (*six6*, *pax2*, *pax6a*, and *pax6b*). (**B**) Expression levels of genes associated with apoptosis (*tp53*, *casp3*, *bax*, and *bcl2a*). (**C**) Expression levels of genes involved in antioxidant defense (*sod1*, *gpx1a*, and *cat*). Data are presented as mean ± SD. Significant differences are denoted by different letters (*p* < 0.05). Each experiment was independently replicated three times.

PSNP exposure led to a significant downregulation of key visual development genes, including *six6*, *pax2*, *pax6a*, and *pax6b*. However, these effects were fully reversed with the addition of VitE (Figure 5A). For apoptotic genes, PSNP exposure resulted in the upregulation of pro-apoptotic genes such as *tp53*, *casp3*, and *bax*, while the anti-apoptotic gene *bcl2a* significantly downregulated. The addition of VitE effectively mitigated these alterations in apoptotic gene expression (Figure 5B). Furthermore, PSNP exposure significantly upregulated genes involved in antioxidant defense, including *sod1*, *gpx1a*, and *cat*, and these increases were also completely reversed by VitE addition (Figure 5C).

## 3. Discussion

The toxic effects of PSNPs have been well documented in various studies, including their cytotoxicity, genotoxicity, hepatotoxicity, neurotoxicity, and reproductive toxicity [36,37]. This study demonstrated that PSNPs may induce visual dysfunction in zebrafish larvae, showing their bioaccumulation in eyes, impaired vision, oxidative stress, apoptosis, and alterations in gene expression. It also highlighted the protective role of VitE in mitigating these harmful effects. The concentration-dependent fluorescence of PSNPs in zebrafish eyes demonstrated their strong affinity for biological tissues and their potential for targeted organ bioaccumulation. These findings align with previous studies reporting NP bioaccumulation in zebrafish eyes [13,38], zebrafish brains [39], sea urchins [40], and marine biota [41]. Additionally, research has shown NP bioaccumulation in zebrafish gills and intestines, resulting not only in localized damage but also systemic toxicity [10]. This highlights the broad and complex toxicological impacts of PSNPs on aquatic organisms. This study advances the understanding of PSNP bioaccumulation, specifically targeting the zebrafish visual system. This observation highlights the eyes as a particularly vulnerable site for NP-induced toxicity, raising concerns about potential impacts on visual system in aquatic life.

The ratio between eye size and body length in zebrafish plays a crucial role in visual development as it directly affects proper eye growth and functionality [29]. In this study, exposure to PSNPs disrupted this ratio, leading to a decreased in eye-to-body length and impaired OMR behavior, which serves as a key indicator of visual performance [42]. These findings align with prior studies showing a proportional relationship between eye size and body size in zebrafish [43]. Similarly, in other species, eye size is tightly regulated relative to body size to maintain visual and physiological balance [44,45]. However, many animal models with eye abnormalities exhibit deviations in body size, further underscoring the interconnectedness of eye development and overall growth [46]. The OMR test has proven to be an effective tool for detecting visual impairments caused by genetic mutations and environmental toxins in zebrafish [47,48]. Consistent with this, a reduced eye-to-body length ratio caused by PSNP exposure was linked to a significant decline in OMR performance in zebrafish [29]. For instance, Le et al. demonstrated that retinoic acid deficiency, which causes microphthalmia, resulted in visual deficits such as reduced optokinetic reflex (OKR) and diminished electroretinogram (ERG) activity [49]. Similarly, studies have demonstrated that genetic or environmental factors that reduce eye size can contribute to the development of myopia and significantly impair visual performance [43]. These findings further indicated that PSNP exposure reduced eye size in zebrafish, adversely affecting visual function and highlighting the importance of proper eye development in maintaining visual capabilities.

Previous studies have established a strong link between reduced eye size and increased retinal cell apoptosis, emphasizing apoptosis as a critical factor in ocular development and function [29,50,51]. In this study, the increased area of AO-positive cells observed in zebrafish exposed to PSNPs suggests that visual dysfunction is primarily driven by enhanced retinal cell apoptosis. PSNPs have also been reported to induce apoptosis in zebrafish cardiomyocytes [52] and skeletal muscle cells [53], as well as in the liver tissue of mice [54], further highlighting their pro-apoptotic effects across multiple systems. Apoptosis, or programmed cell death, is a critical mechanism underlying the retinal damage observed in this study. The upregulation of pro-apoptotic genes such as *tp53*, *casp3*, and *bax*, along with the downregulation of the anti-apoptotic gene *bcl2a*, indicated that PSNPs induced apoptosis through intrinsic pathways. The upregulation of *tp53* suggests that DNA damage induced by ROS triggered apoptotic pathways [55], consistent with prior studies on oxidative-stress-mediated apoptosis [29]. The activation of BCL-2 family members *bax* and *bak*, along with caspase-3, is a crucial step in the intrinsic apoptosis pathway [56]. This process leads to mitochondrial outer membrane permeabilization, a pivotal event that initiates the cascade of apoptotic signaling and cellular dismantling [57]. Additionally, the downregulation of the anti-apoptotic gene *bcl2a* highlights the imbalance between pro- and anti-apoptotic signals, promoting cell death [58]. These findings confirmed that apoptosis is a major consequence of PSNP exposure and highlight its role in the retinal damage and visual dysfunction observed in zebrafish larvae.

While ROS play essential roles in cellular signaling and defense mechanisms [59], excessive ROS production can lead to oxidative stress, causing damage to lipids, proteins, and DNA [60]. Exposure to PSNPs has been shown to trigger ROS production, resulting in intestinal cytotoxicity in *C. elegans* [61]. ROS production is a well-documented mechanism of NP toxicity [62]. In this study, PSNP exposure significantly increased ROS levels in a concentration-dependent manner, particularly in the retina of zebrafish larvae. This suggests that oxidative stress is a primary driver of PSNP-induced toxicity. NPs have been reported to cause oxidative damage and inflammation in cells, activating the p38 MAPK signaling pathway and leading to cell apoptosis [63]. Elevated ROS levels overwhelmed the antioxidant defense mechanisms [64], as evidenced by the upregulation of key oxidative stress response genes such as *sod1*, *gpx1a*, and *cat after PSNP exposure*. These genes are vital for mitigating oxidative damage: *sod1* produces superoxide dismutase to convert superoxide radicals into hydrogen peroxide, *gpx1a* encodes glutathione peroxidase to reduce hydrogen peroxide to water, and *cat* encodes catalase to detoxify hydrogen peroxide [65,66]. The overwhelming ROS production observed suggests that endogenous antioxidant systems are insufficient to mitigate the damage [29,67].

Exposure to PSNPs significantly disrupted the expression of key genes essential for visual development, including *six6*, *pax2*, *pax6a*, and *pax6b* [68]. These genes are critical for retinal differentiation, photoreceptor development, and optic nerve formation, which are fundamental to maintaining proper visual function [69,70]. *Six6* regulates opsin genes such as *SWS2* and *RH2*, which are vital for zebrafish vision by enabling color discrimination and light sensitivity. These opsins are predominantly expressed in the eyes throughout both larval and adult stages [70]. In *pax6*-knockout mice, significant reductions in V1 neurons were observed, demonstrating the pivotal role of *pax6* in locomotion and visual–motor integration through its regulation of V1 neuron activity [71]. Similarly, in zebrafish, *pax6* has been shown to be indispensable for eye development, playing a critical role in the formation and function of ocular structures [69]. Recent studies further highlight the importance of *pax6a* and *pax6b* in visual function, with their reduced expression directly linked to visual dysfunction in zebrafish embryos [29] and adults [30]. These visual development genes were downregulated in this study, possibly causing physiological and functional defects in the zebrafish retina. These findings highlight the detrimental impact of PSNP exposure on the visual system, particularly its role in disrupting critical molecular pathways essential for proper visual development and function.

The addition of VitE effectively mitigated the adverse effects of PSNPs highlighting its potential role in reducing oxidative stress and associated damage. VitE restored the expression of antioxidant genes and significantly reduced ROS levels, thereby reversing oxidative damage and apoptosis in retinal cells. Its ability to combat oxidative stress has been well documented, with studies showing that VitE provides substantial benefits in reducing oxidative stress levels [72]. Previous research further supports the protective role of VitE in mitigating retinal damage. For instance, VitE has been shown to reduce radiation-induced cataract formation in rats [73] and protect against retinal injury in guinea pigs [5]. Additionally, various forms of VitE have demonstrated protective effects during retinal ischemia–reperfusion injury, effectively preventing cell damage and preserving retinal function [1]. VitE’s efficacy in ocular protection may also be attributed to its naturally higher concentrations in all ocular tissues [2,3], suggesting a critical role in maintaining visual system integrity. Furthermore, cases of VitE deficiency have been linked to visual defects, reinforcing its importance for eye health [74]. While the precise mechanisms underlying VitE’s protective effects require further investigation, these findings highlight its diverse roles in counteracting oxidative stress and cellular damage, especially in the retina. This research highlights its potential efficacy in mitigating the detrimental effects of PSNPs and other environmental stresses on visual health.

Overall, this study comprehensively examined toxicological impacts of PSNPs on the zebrafish visual system, demonstrating their capacity to induce oxidative stress, disrupt essential developmental genes, and trigger retinal cell apoptosis. The bioaccumulation of PSNPs in ocular tissues caused structural damage and impaired visual function, raising broader concerns about the effects of NPs on aquatic organisms and ecosystems. Importantly, this research highlighted the role of VitE in mitigating these toxic effects. VitE effectively reduced ROS levels, restored antioxidant defenses, and mitigated cellular damage in the zebrafish visual system. Additionally, the results emphasized the ecological risks posed by NP pollution. PSNPs bioaccumulated in tissues, causing both localized and systemic toxicity, which threatened not only zebrafish but also other aquatic species. This disruption had the potential to harm biodiversity and destabilize ecosystems. By advancing our understanding of PSNP toxicity and demonstrating the mitigating role of VitE, this research provided valuable insights into addressing nanoparticle pollution in aquatic environments. The study addressed the importance of developing practical strategies, such as antioxidant supplementation, to protect aquatic ecosystems and maintain ecological balance. However, further study is necessary to investigate the scalability, feasibility, and long-term implications of these interventions in natural aquatic environments, where complexities such as environmental variability and interactions with other stressors must be considered.

## 4. Materials and Methods

### 4.1. Maintenance of Zebrafish

Wild-type zebrafish (*Danio rerio*) were obtained from Taiwan Zebrafish Core Facility at Academia Sinica (Taipei, Taiwan) and maintained in 40-L tanks at a stable temperature of 28 °C under a controlled light cycle of 14 h of light and 10 h of darkness. They were fed daily with commercial pellets and supplemented with brine shrimp twice a day at 9:00 am and 5:00 pm. For breeding, adult males and females were placed in mating boxes at a 2:1 ratio, separated by a partition for acclimatization. The following morning at 9:00 am, the partition was removed, allowing mating under light stimulation. Eggs were collected 30 min after spawning and washed with circulating system water to eliminate dead embryos and debris. Fertilized eggs were then rinsed with embryo medium (EM) containing 0.004% CaCl_2_, 0.163% MgSO_4_, 0.1% NaCl, and 0.003% KCl. The cleaned embryos were evenly distributed into 6-well plates, with 50 embryos per well in 8 mL of EM, and maintained at 28 °C. All zebrafish procedures complied with local animal welfare regulations and were approved by the Institutional Animal Care and Use Committee (IACUC) of NPUST on 19 August 2024.

### 4.2. Exposure Experiments

Red fluorescent PSNPs were purchased from Thermo Fisher Scientific (Waltham, MA, USA) have been previously characterized for their physicochemical properties [75,76]. Transmission electron microscopy (TEM) analysis with negative staining confirmed their round shape and size, consistent with the manufacturer’s specifications. The PSNPs exhibited an average size of 25 ± 0.6 nm [75]. These properties remained stable upon dilution with distilled water [76], supporting its suitability as the diluent in this study. Stock solutions of PSNPs were diluted with distilled water to prepare experimental concentrations of control (0 μg/mL), 0.01, 0.1, 1, and 10 μg/mL. These concentrations were selected based on a pilot study (unpublished) to evaluate the biological effects of PSNPs and reflect reported environmental levels of up to 10 μg/mL [77]. To ensure uniform dispersion and prevent aggregation, PSNP solutions were sonicated for 3–5 min before use.

Vitamin E (VitE; D-α-Tocopherol polyethylene glycol 1000 succinate) was obtained from Sigma-Aldrich (Merck, Darmstadt, Germany). A 100 mM stock solution was prepared following the method of Zhang et al. [78] using dimethyl sulfoxide (DMSO) and Tween-80 as solvents. Final concentrations of DMSO and Tween-80 in the exposure solutions were limited to 0.0019% and 0.000010% (*v*/*v*), respectively, to ensure they remained non-toxic to zebrafish embryos. A concentration of 20 μM VitE was selected based on prior studies reporting its protective effects in zebrafish without toxicity [78]. Zebrafish embryos were exposed to PSNPs with or without 20 μM VitE simultaneously from 2 h post-fertilization (hpf) to 144 hpf. This experimental design allowed an evaluation of the combined and cumulative effects of PSNPs and VitE during the developmental period. For control groups, the exposure media contained only 0.0019% DMSO and 0.000010% Tween-80, matching the experimental groups. Exposures were conducted in 6-well plates, with 50 embryos per well and three replicates per experimental group, using eggs from different spawns. The embryos were maintained at a constant temperature of 28 °C, and exposure solutions were refreshed daily to ensure consistency throughout the study.

### 4.3. Properties of PSNPs

According to the manufacturer, the fluorescent PSNPs (25 nm in size) were obtained from Thermo Fisher Scientific Co. (Waltham, MA, USA) (Catalog Number: R25). These NPs consist of 1% solid particles labeled with a red fluorescent dye, featuring excitation and emission wavelengths of 542 nm and 612 nm, respectively. With a density of 1.06 g/cm³, the particles are suspended in deionized water containing trace amounts of surfactants and preservatives to ensure stability and prevent particle aggregation. The fluorescent label used in these PSNPs is commercially available and widely utilized in biological experiments, with minimal reported toxicity [75,76]. Recent studies have comprehensively characterized these PSNPs, including their shape, size, polydispersity index (PDI), and ζ-potential. TEM analysis with negative staining confirmed their round shape and size, which are consistent with the manufacturer’s certificate of analysis. Furthermore, it was demonstrated that dilution with distilled water did not significantly alter their shape, size, or tendency for aggregation [76]. Based on these findings, distilled water was used in this study to ensure the consistency, reproducibility, and accurate interpretation of the biological effects of PSNPs.

### 4.4. Bioaccumulation of PSNPs in Zebrafish Eye

The bioaccumulation of PSNPs in the zebrafish eye was evaluated using fluorescence imaging. Zebrafish larvae were exposed to fluorescently labeled PSNPs at concentrations of 0.01, 0.1, 1, and 10 µg/mL, and the bioaccumulation in the eyes was assessed at 96 hpf. Following the exposure process, larvae were thoroughly rinsed three times with distilled water to remove any non-internalized or surface-adhered PSNPs. For imaging, larvae were anesthetized in 0.003% tricaine solution and mounted in 0.03% agarose. Fluorescent signals were captured using a red fluorescence microscope (Leica M165 FC, Wetzlar, Germany), with consistent exposure settings applied across all groups to ensure comparability. PSNP bioaccumulation in the eyes, indicated by red fluorescence, was quantified as the area of PSNP bioaccumulation focused on the eye region using ImageJ 1.53i software. Brightness and contrast settings were standardized based on the fluorescent signal from the 10 µg/mL PSNP group to maintain uniformity across samples. Results were expressed as fold changes relative to the control group, providing an effective quantification of PSNP bioaccumulation.

### 4.5. Eye and Body Length Measurement

Zebrafish larvae at 96 hpf were selected for eye-to-body length measurements as this stage marks significant eye maturation, including retinal differentiation, photoreceptor development, and optic nerve formation [23]. Measurements were conducted following established protocols [43]. The eye diameter was determined as the distance between opposite poles of the pigmented epithelium, while body length was measured from the snout to the end of the spine, excluding the caudal fin. All measurements were performed from a lateral view under a microscope (Leica M165 FC, Wetzlar, Germany) and converted into millimeters (mm). Each experimental group consisted of ten embryos, and the procedure was repeated three times with larvae from independent spawns.

### 4.6. Visual Behavior Assay

The OMR assay was used to assess visual behavior in zebrafish larvae, focusing on their head and body movements in response to visual stimuli [79]. At 6 dpf, zebrafish larvae have developed sufficient motor coordination, making it an optimal stage for assessing visual and behavioral responses [80]. This method was adapted from a previously published protocol [29], which involved placing 6 dpf larvae in a custom petri dish with five parallel tracks (0.5 cm × 7 cm) filled with distilled water. The setup was placed on a white screen under 825 nm light. The 825 nm wavelength was chosen because it avoids interference with the larvae’s visual perception, which predominantly operates within the visible spectrum [81]. The testing environment was kept in complete darkness to eliminate external light interference, and the temperature was consistently maintained at 25–28 °C to prevent thermal stress. These controls ensured reliable and consistent results across all trials. After 30 s of acclimation, zebrafish were exposed to moving black-and-white bars as a stimulus, and their responses were recorded for 30 s before and after exposure. Two measurements were recorded: the number of larvae showing positive OMR by following the stimulus direction and their swimming distance over 30 s. Swimming distance was measured manually using ImageJ 1.53i software. Each group was examined with ten embryos, and the experiments were repeated three times with larvae from independent spawns.

### 4.7. Apoptosis Cell Assay

Retinal apoptosis was evaluated using AO staining at 96 hpf [29]. Larvae were incubated in a solution of 5 µg/mL acridine orange (acridinium chloride hemi-[zinc chloride], Sigma-Aldrich, Darmstadt-Germany) dissolved in embryo medium (EM) for 30 min at 28 °C in darkness to prevent the photo-degradation of the dye. After staining, embryos were thoroughly rinsed with EM to remove excess AO, anesthetized with 0.016% tricaine, and carefully positioned laterally on slide glass using 0.5% gel agarose for stability. Apoptotic cells in the retina were visualized under a fluorescent microscope (Leica M165 FC, Germany), focusing specifically on the eye region. Areas of apoptosis were identified by AO-positive fluorescence, and fluorescence intensity was quantified using ImageJ 1.53i software with a standardized thresholding protocol to reduce subjective bias. Each experimental group included ten embryos, and the experiment was repeated three times with larvae from independent spawns.

### 4.8. ROS Production

To measure ROS levels, ten larvae at 96 hpf were randomly selected and analyzed using chloromethyl-20,70-dichlorodihydrofluorescein diacetate (CM-H2DCFDA, Invitrogen, Waltham, MA, USA) [82]. This reagent serves as a broad indicator of oxidative stress by reacting with ROS, which oxidize CM-H2DCFDA into its fluorescent form, DCF. The larvae were washed three times with ultra-pure water, incubated in 1 μg/mL CM-H2DCFDA solution for two hours in the dark, and washed again post-incubation. They were then immobilized in 0.3% methylcellulose. Fluorescent images of live samples were obtained using a stereomicroscope, and ROS fluorescence intensity was analyzed using ImageJ 1.53i software. The procedure was repeated three times with larvae from independent spawns.

### 4.9. Real-Time PCR

Zebrafish larvae were exposed to 0.1 µg/mL PSNPs with or without 20 µM VitE, and total RNA was extracted at 96 hpf (*n* = 20 per treatment group). Quantitative PCR was utilized to assess the expression levels of six homeobox 6 *(six6)*, pair box protein (*pax2, pax6a,* and *pax6b*), tumor protein 53 (*tp53*), caspase 3 (*casp3*), BCL2-associated X, apoptosis regulator a (*bax*), b-cell leukemia/lymphoma 2α (*bcl2a*), superoxide dismutase-1 (*sod1*), catalase (*cat*), glutathione peroxidase 1a (*gpx1a*), and elongation factor 1 alpha 1 (*eef1a1*), determined using quantitative PCR. The *eef1a1* was used as an internal control. The specific primers used in this experiment are detailed in Table 1. Real-time PCR was carried out using KAPA SYBR FAST PCR reagent (KAPA Biosystems, Wilmington, MA, USA) and an Applied Biosystems StepOnePlus Real-Time PCR system (Thermo Fisher Scientific, Foster City, CA, USA). The cycling profile included enzyme activation at 95 °C for 3 min, denaturation at 95 °C for 3 s, followed by annealing/primer extension for 40 cycles with denaturation at 95 °C for 3 s.

### 4.10. Statistical Analysis

Data were initially assessed for normality using the Shapiro–Wilk test and for the homogeneity of variances using Bartlett’s test to ensure the validity of subsequent analyses. Statistical comparisons were conducted using one-way ANOVA, followed by Tukey’s post hoc test to identify significant differences among treatment groups. All analyses were performed using SigmaPlot 12.5 software for Windows, with a significance threshold set at *p* < 0.05. Results are reported as mean ± standard deviation (SD).

## Figures and Tables

**Table 1:** Oligonucleotides sequences used for gene expression analysis.

	Gene	Forward Sequence (5’-3’)	Reverse Sequence (3’-5’)
Visual System	*six6*	CGAACTCGCGGTTTGTTGAG	CGTGATGCTGAAGCCTGTTTT
	*pax2*	CCCGCGTTATTAAGTTCCCC	GATGTCCGCTGTTGCTTGAC
	*pax6a*	CTCAAACAGAAGAGCGAAATGGA	GAAGCTGCTGCTGATGGGTAT
	*pax6b*	CCTCCAGTCACATTCCCATCA	AGCATTGAGCCTGTCGTGAA
Apoptosis	*tp53*	GGGCAATCAGCGAGCAAA	ACTGACCTTCCTGAGTCTCCA
	*casp3*	CCGCTGCCCATCACTA	ATCCTTTCACGACCATCT
	*bax*	CCGTGAGATCTTCTCTGATGG	GTCAGGAACCCTGGTTGAAA
	*bcl2a*	AGGAAAATGGAGGTTGGGATG	TGTTAGGTATGAAAACGGGTGGA
Antioxidant	*sod1*	GTCGTCTGGCTTGTGGAGTG	TGTCAGCGGGCTAGTGCTT
	*gpx1a*	GGCACAACAGTCAGGGATTA	CAGGACGGACGTATTTCAGA
	*cat*	TACCAGTCAACTGCCCGTAC	GACTCAAGGAAGCGTGGC
	*eef1a1*	TGGTGGTGTCGGTGAGTTTG	AAACGAGCCTGGCTGTAAGG

## Data Availability

The data obtained in this study are available upon reasonable request from the corresponding author.
